# Time is on My Side—or Is It? Assessing How Perceived Control of Time and Procrastination Influence Emotional Exhaustion on the Job

**DOI:** 10.3390/bs10060098

**Published:** 2020-06-10

**Authors:** Catherine A. Roster, Joseph R. Ferrari

**Affiliations:** 1 Anderson School of Management, The University of New Mexico, Albuquerque, NM 87131, USA; 2 Department of Psychology, DePaul University, Chicago, IL 60614, USA; jferrari@depaul.edu

**Keywords:** job stress, job demand–control model, emotional exhaustion, procrastination, perceived control over time, mediation analysis, moderated mediation analysis

## Abstract

The job demands–control model (JDC) postulates that an increased control over work resources mitigates or “buffers” the positive association between job stressors and strainers. However, the inconclusive validation of the buffering hypothesis across multiple studies suggests the need for fresh approaches, both conceptual and methodological. We integrated aspects of the JDC framework and time management process models to construct a model that tested both the direct and indirect effects of the perceived control of time (PCT) on emotional exhaustion arising from workload demands. Furthermore, we tested whether procrastination tendencies moderated the benefits of PCT on work stressors and strains. Data were collected in an Internet survey with 356 US adult office workers obtained from Prolific. The results supported the buffering effect of PCT on emotional exhaustion. PCT both mediated and exerted direct effects on the relationship between workload and emotional exhaustion. Procrastination tendencies moderated PCT and, in turn, undermined high PCT ability to reduce emotional exhaustion. Overall, the findings suggested that giving workers more control over their time may reduce stress associated with demanding workloads. However, chronic procrastinators may benefit less from having more control over time resources if they are not provided with tools to help them self-regulate more effectively.

## 1. Introduction

The nature of work has changed dramatically over the past several decades, responding to economic implications of globalization, the rise of e-commerce, and rapid advancements in technology [1,2]. As organizations downsize, restructure, automate, and computerize tasks, the psychological demands placed on workers at all levels of the organization continue to rise. A 2017 survey of 1500 USA full-time, part-time, and self-employed workers conducted for the American Psychological Association by Harris Polling found that workers undergoing workplace changes were twice as likely to experience chronic stress as workers undergoing no change, and four times as likely to have physical health symptoms [3]. The same study also found that younger workers between the ages of 18 and 36 were more likely than workers between the ages of 37 to 52 or workers in the oldest age group to report that they were experiencing stress and physical symptoms during the workday. Jobs are also more complex than they were in the past, requiring workers to learn new skills quickly and manage their time effectively in order to meet the rise in work intensity and tight deadlines [2,4]. According to 2019 data from the National Institute for Occupational Safety and Health (NIOSH), 75% of American workers think that they are experiencing more stress than previous generations [5].

One of the most influential models for conceptualizing the relationship between working conditions and workers′ well-being is Karasek′s job demand–control (JDC) model [6,7]. The model identifies two factors—job demands and job control—that impact workers′ well-being. In the JDC model, job demands are typically associated with the pace and volume of work, while job control reflects a worker’s ability to exert control over the work environment. Two general and interrelated hypotheses propose how these factors operate together to predict outcomes for workers′ health. First, the job ‘strain’ hypothesis predicts that high demands combined with low control lead to negative outcomes on workers′ health and well-being. Second, the ‘buffer’ hypothesis proposes that high control over resources can mitigate the negative effects of high demands on workers′ well-being. Prior research has extensively investigated the relationship between job characteristics and job demands and strains and has found strong support for the strain hypothesis [8]. However, the findings regarding the buffer hypothesis have been less consistent [8,9,10]. Researchers have speculated that the inability to show significant buffering effects between job demands and control might be due to a mismatch between the specific job demand and job control variables measured in the study [8,9]. Others have questioned the interpretation of Karasek′s model to mean that an interaction effect must occur between job demands and control variables [10], which Karasek himself clarified in subsequent publications was not necessary [11]. Another explanation that has been offered is a failure to consider the conditional variables—such as individual differences—that might inhibit or exacerbate the buffering effects on job strain [12,13,14].

A related perspective of how work demands are controlled by workers is found in Macan′s process model of time management [15]. This model proposes that planning behaviors are positively associated with perceived control of time (PCT), which, in turn, mediates the relationship between planning and positive outcomes, such as higher job performance, satisfaction, and reduced job strain. Claessens et al. [16] conducted a test of Macan′s model that included workload demands as an independent variable along with planning behaviors and found support for PCT as a partial mediator for the job demands–job strains relationship. This suggested that PCT could operate as a buffer for high job demands in the JDC model, and prompted the study’s authors to recommend that future research examines the potential mediating role of PCT in the JDC model more closely [16] (p. 947).

The purpose of the present study was to investigate the mitigating influence of the perceived control of time (PCT) on emotional exhaustion arising from heavy workload demands. We collected data from a sample of 356 US office workers purchased from Prolific, who completed an Internet questionnaire created with Qualtrics. We tested our hypotheses in a moderated mediation model, in which we posited that PCT mediated the positive relationship between job demands and emotional strain. We predicted that workers with higher levels of PCT would feel less emotionally exhausted by heavy workloads than workers with lower levels of PCT. Additionally, because PCT is a time-oriented variable, we predicted that workers with a greater tendency to procrastinate would not benefit as much by having more control over their time as those less likely to procrastinate.

The current study addresses current gaps in the literature and expands the framework for investigating the impact of control variables on job stressors and outcomes. We examined a specific control variable (PCT) matched to a particular stressor (workload demands and time pressures). Another contribution of this work is the integration of the JDC model with time management process models. Our study provides further support for integrating these two theoretical models and treating PCT as a buffer control variable. Furthermore, our study is the first to incorporate procrastination tendencies as an independent difference variable that potentially moderates workers′ PCT and, subsequently, the PCT′s mitigating influence on the work demands–emotional exhaustion relationship.

## 2. Theoretical Framework and Hypotheses

### 2.1. Stressors and Strain in the JDC Model

At the core of the JDC model is the relationship between job stressors (i.e., demands) and job strains (i.e., reactions to stressors). Karasek defined job demands as “the psychological stressors involved in accomplishing the workload” [6] (p. 291). The most common “stressor” examined in the JDC model is workload demands, which are typically operationalized in terms of the volume of work required of an employee, the amount of work to be accomplished in a set period of time, or the mental demands of the work being performed [17]. Other stressors, while less commonly examined, include organizational constraints, such as inadequate information leading to role and task ambiguity [8,10,14,18], and psychosocial stressors, such as work–family conflicts [19] or conflicts with supervisors [20]. In terms of job strains, researchers have examined a broad array of the psychological and job-related measures of well-being, including depression, anxiety, role stress, burnout and emotional exhaustion, job satisfaction, motivation, and turnover [8,10,21], as well as a variety of physical health outcomes, including cardiovascular disease, all-cause mortality, psychosomatic illnesses, gastro-intestinal disease, musculoskeletal pain, fatigue, and negative pregnancy outcomes [21,22].

According to Karasek′s JDC model [6], the outcomes of a worker′s psychological or physical well-being are a function of both the job demands and job control. Karasek defined job control as a “working individual′s potential control over his task and his conduct during the working day” [6] (pp. 289–290). The model offers two central hypotheses with respect to how job demands and job control combine to create alternative outcomes. The “strain hypothesis” predicts negative outcomes for workers in high-strain job situations, which occur when demands are high and workers′ control is low. The other central hypothesis is the “buffer” hypothesis, which predicts that the detrimental effects of high demands on workers′ well-being are mitigated by increased job control. The two hypotheses are not mutually exclusive, as researchers have noted [8], in that the buffer hypothesis is essentially a specification of the more general strain hypothesis, not a separate hypothesis predicting a fundamentally different outcome. Instead, Karasek conceptualized learning, motivation, and the development of new skills as positive outcomes predicted by an opposing diagonal from that of strain outcomes [23]. In his “active learning” hypothesis, workers facing high demands in contexts where they are given high control or job autonomy may over time come to view stressors as positive “challenges” that offer opportunities to learn and grow, as long as the psychological demands are not “too overwhelming” [23] (p. 34–37).

Research has found considerable support for the strain hypothesis. Van der Doef and Maes [8] conducted a review of studies investigating the JDC model strain hypothesis from 1979–1997. They found consistent support, especially in studies involving larger samples with general populations of mainly blue-collar workers, for the predictions associated with strain and psychological well-being outcomes. Altogether, the findings from multiple studies demonstrated that working in a high-strain job with low control appears to be associated with lower general psychological well-being, lower job satisfaction, more burnout, and more job-related psychological distress. With regards to four studies that investigated burnout, all but one study supported the main effects of the strain hypothesis, but none supported the buffering effect of control as a moderator. Häusser et al. [10] reached similar conclusions following an updated review of studies published between 1998 and 2007, especially for the main effects of JDC dimensions on psychological well-being and for additive effects of demands and control on emotional exhaustion. No significant differences were found regarding gender, sample composition, application of measurement instruments, or job-related homogeneity for emotional exhaustion. Only two of the studies in the more recent review conducted by Häusser et al. [10] examined the buffer hypothesis of the JDC model—one of which found support for additive, but not multiplicative effects of demand and control variables. 

In the current study, the stressors were operationalized in terms of workload and time pressures and psychological strain was operationalized as emotional exhaustion. Based on prior research that has found wide-spread support for the strain hypothesis across multiple contexts and samples, we posit the following hypothesis:

**Hypothesis** **1.** *Workload demands will be positively related to emotional exhaustion*.

### 2.2. The Buffer Hypothesis and Resource Control 

The buffer hypothesis predicted by the JDC model posits that control over the work environment may buffer—or mitigate—the potentially negative effects of high job demands on workers′ health and emotional well-being [5,7]. In the organizational psychology literature, control is often conceptualized as workers′ overall influence or their control over specific task characteristics [9,21]. In a meta-analysis of studies examining worker participation in decision-making and job autonomy, Spector found that high levels of perceived control were associated with a variety of positive outcomes, including higher levels of job satisfaction, commitment, involvement, performance and motivation, and lower negative outcomes, including physical symptoms, emotional distress, absenteeism, and turnover [21]. Spector′s findings were based on average correlations across study samples, not the multiplicative or interaction effect between job demands and control that some researchers regard as integral to the buffer hypothesis [8,9,22]. 

Karasek′s initial conceptualization of job control was narrowly limited to decision latitude, although he acknowledged the need to consider a wider range of resources, including task organization, time pacing, influence over organizational policies, and skills [5] (p. 290). Later, Ganster provided a more flexible definition of control as “the ability to exert some influence over one′s environment so that the environment becomes more rewarding or less threatening” [9] (p.3). He further distinguished between the objective presence of control and an individual′s perception or belief in that control, concluding that the latter was more likely to directly influence mental and physical health outcomes. Ganster [9], citing the failure of most studies to find support for the buffer hypothesis in tests of significant interaction effects between demand and control variables, recommended a deeper look into how control has been conceptualized and assessed in the literature. He suggested that specifying control over the particular stressor of concern to the individual might potentially be more useful for alleviating distress than the current practice, which is to measure control with broad constructs, such as “job autonomy” or “worker participation.”

The particular stressor considered in the current study is workload demands created by heavy workloads with fast work pace. Perceived control of time (PCT) is a construct used in time management literature to reflect “an employee′s perception of having enough time to finish one′s work and the ability to meet one′s deadlines” [24] (p. 430). Process models of time management conceptualize PCT as a mediator between time management behaviors, such as goal setting, planning, and monitoring activities, and job outcomes, including job satisfaction, well-being, and job performance [15,16]. Studies have shown that PCT at least partially mediates the relationship between time management variables and outcomes for well-being and job satisfaction [15,16,19]. Claessens et al. [16] found that PCT mediated the relationship between workload demands and work strain, leading them to conclude that incorporating time management variables, such as PCT, in the JDC model could provide insights into the work stressor and strain process. Thus, we propose that workload demands in the form of high volumes of work with time pressures will be directly and negatively related to PCT.

**Hypothesis** **2.** *Workload demands will be negatively related to PCT*.

### 2.3. Conditional Mediating Role of PCT

PCT′s effectiveness in mitigating mental job strain may be contingent upon a worker′s ability to allocate time resources effectively and meet deadlines. Procrastination is a behavioral disposition where people postpone or delay tasks that consequently lead to performance failures and increased task anxiety [25,26]. Procrastination is often linked to the construct of PCT, but has not been investigated as a separate individual variable that could impact job control and demand outcomes. When Macan, Shahani, Dipboye, and Phillips [27] first introduced the PCT variable into time management literature, they suggested that procrastination was a facet of PCT, as it affects an individual′s perception of available time. The current predominant view of procrastination is that it represents a maladaptive intention–action gap arising from self-regulation failure [28,29]. Although some scholars have proposed an adaptive form of procrastination called “active procrastination” that can produce positive performance outcomes [30], research consistently demonstrates that low self-regulation is the trademark characteristic of procrastination and that it leads to negative outcomes [25,27,28], including performance failures [31,32] and heightened stress levels [33,34]. In the time management literature, studies have shown that productive time management behaviors lead to higher PCT, which, in turn, reduces job stress [16,24]. Therefore, according to Macan′s time management model [15], when individual choices represent an ineffective use of time, such actions should negatively influence the extent to which workers feel in control of their time, therefore undermining the PCT′s ability to help workers feel less strained and more productive in their work. Hypothesis 3 expresses our expectation that procrastination tendencies will moderate the impact of PCT on office workers′ emotional exhaustion.

**Hypothesis** **3.** *Procrastination tendencies interact with PCT, so that the mitigating (i.e., inverse) impact of PCT on emotional exhaustion is less when a worker′s procrastination tendencies are high*.

The proper way to statistically test the theoretical influence of control variables in the JDC model has been a source of debate among organizational psychology researchers. Most researchers regard the moderating influence of control on demand and outcome variables as the crucial test for the buffer hypothesis, although empirical studies have not provided convincing support for interaction effects across different research contexts [8,10]. Karasek [11] argued that multiplicative interactions between job demand and job control variables are not necessary, as the model was designed to predict different outcomes (i.e., strain or learning) based on workers′ active or passive responses to different workplace environments. In her review of the theories used to explain the relationship between control and stress responses, Miller [35] also suggested that control can be a function of both direct and indirect combinations of factors. Drawing upon Seligman′s notions of instrumental control [36], Miller proposed that individuals might actively or passively draw upon their perceived internal ability to exert control over circumstances as a means of mitigating or reducing the severity of environmental stressors. According to her theoretical proposition, which she termed, “the Minimax hypothesis,” workers might find high demands under their control less subjectively stressful than low demands not under their control. This is because controllability provides additional predictability, and the predictability of adverse effects has been known to reduce stress responses [37]. When an individual attributes the source of control resources to internal—as opposed to external—sources, it becomes more stable and, therefore, predictable. Miller asserts that the source of control is important to consider and may help to explain conflicting findings in the control and stress literature.

The characteristics of the job, such as job autonomy and participation, represent external sources of control. In contrast, PCT, depending on how effectively a worker utilizes their time resources and responds to time pressures, is within the control of the individual. Based on this notion and support in the time management literature for the PCT′s role as a mediator of the relationship between workload demands and work stress [16], we posit our prediction for a moderated mediation influence of PCT and procrastination tendencies on the relationship between workload demands and emotional exhaustion in Hypothesis 4.

**Hypothesis** **4.** *PCT mediates the positive relationship between workload demands and emotional exhaustion and the strength of the mediation is contingent upon the moderating effect of a worker′s procrastination tendencies*.

**Figure 1:**
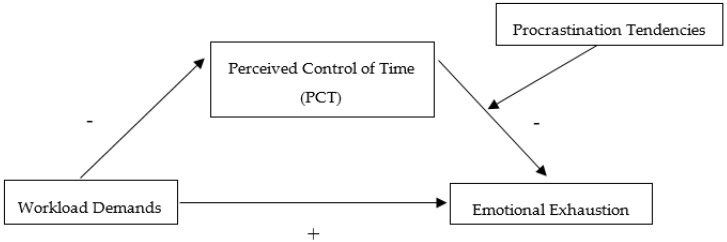
The proposed moderated mediation model.

Figure 1 represents a conceptual model of the proposed moderated mediation model we used to test our study hypotheses.

## 3. Research Methodology

### 3.1. Sample and Procedures

We collected data using an Internet survey created with Qualtrics (www.qualtrics.com) survey software. A convenience sample of 400 respondents were recruited from Prolific (www.prolific.co), a UK-based crowdsourcing platform for connecting researchers and participants from around the world. Prolific prescreens respondents registered on their platform based on sample eligibility requirements requested by the researcher. Eligibility requirements for this study included: (1) resident of the United States; (2) between the ages of 21 to 75; (3) employed for more than 20 h per week; and 4) performs work duties in a traditional office workspace designated for their personal use. Each respondent who completed the survey was compensated $2.50 for his/her participation. On average, it took the respondents 15 min to complete the survey.

Three hundred and ninety-eight respondents completed all questions. Thirty-eight of the 398 respondents who completed the survey indicated that they did not work at least 20 h per week from a traditional office workspace and were excluded from further analysis, leaving 360 eligible respondents. Slightly more than half of our sample were male (53%). The median age group was 25 to 35 (55%), which is younger than the general US population. The median income group was $35,000 to $49,999 and most (42%) reported having a bachelor′s degree. The sample was predominately Caucasian (76%). The average hours worked per week was 39.6 h. The average length of employment in the current job was 3 years. Most respondents classified their job as being staff/administrative (36%) or individual contributors (i.e., “other” non-management employees) (28%). Eleven percent equally self-classified themselves as either top, middle, or lower management (33% collectively) and three percent chose “I′d prefer not to answer.” In terms of their positions, 7.3% reported their position as executive or managerial, 10% were entrepreneurs or owners, 39% worked in professional or technical services, 4% reported being in sales, 17% worked in clerical or administrative support roles, 8% were in customer service, 11% reported “other” (e.g., self-employed contractor, freelancer, educator, legal, etc.), and 4% responded “I′d prefer not to answer.” Overall, our sample was younger, more highly educated, and less ethnically diverse than the general US population, which is typical of panel samples [38].

### 3.2. Measures

The survey included multiple items, including measures for the variables central to the hypotheses we tested in the current study. All measures relied on widely used and previously validated scales for measuring the four primary variables in our conceptual model (see Figure 1). Below is a description of the scale used to measure each variable. A complete listing of scale items for each of the four primary variables in our model appears in Appendix A.

#### 3.2.1. Workload Demands

We measured workload demands with the five-item quantitative workload inventory (QWI) developed by Spector and Jex [17]. The QWI assessed the amount of work and work pace. The responses were fashioned on a five-point scale, ranging from “less than once per month or never” (1) to “several times per day” (5). The sample questions included: “How often does your job require you to work very fast?” and “How often does your job require you to work very hard?” The Cronbach′s alpha was 0.85.

#### 3.2.2. Perceived Control of Time

Perceived control of time (PCT) has been measured various ways in the time management literature. Since our hypotheses were based on the potential mediating role of PCT in the JDC model, as investigated by Claessens et al. [16], we measured PCT in our study with the same five items. The sample questions included “I feel in control of my time,” and “I feel confident in that I am able to complete my work on time.” All items were measured on a 5-point Likert scale ranging from “strongly disagree” (1) to “strongly agree” (5). The Cronbach′s alpha was 0.79.

#### 3.2.3. Procrastination Tendencies

Procrastination tendencies were measured using the revised adult inventory of procrastination (AIP) scale [18]. The AIP is a well-known psychometric inventory that measures individuals′ behavioral tendencies to delay either beginning or completing tasks. The original 15-item scale asks respondents to rate their tendency to procrastinate with statements such as “I don′t get things done on time,” and “I am not very good at meeting deadlines” on a 5-point Likert scale ranging from “strongly disagree” (1) to “strongly agree” (5). We used a shorter 10-item version of the AIP designed by one of the original scale authors [39]. The Cronbach′s alpha for the 10-item scale was 0.89.

#### 3.2.4. Emotional Exhaustion

Emotional exhaustion was measured with eight items from the emotional exhaustion subscale by Maslach and Jackson in the Maslach burnout inventory [40]. The responses were given on a seven-point scale, ranging from “strongly disagree” (1) to “strongly agree” (7). The Cronbach′s alpha was 0.93.

#### 3.2.5. Control Variable 

Self-report measures of procrastination are known to be associated with social desirability bias [41]. Therefore, we controlled for social desirability response bias using the 13-item short version (Form C) of the Marlow–Crowne social desirability scale [42].

## 4. Results

### 4.1. Data Preparation and Preliminary Data Analyses

We used SPSS v26 including PROCESS macro version 3.1 by Hayes (www.afhayes.com) to analyze our data. PROCESS performs ordinary least squares regression-based path analyses for mediation and moderation effects, including conditional process models, in which the path between the predictor and outcome variable is mediated by an intermediary variable (M) that is moderated by or conditioned on one or more variables [43]. Our conceptual model, as shown in Figure 1, presents a moderated mediation path model that we tested with PROCESS Model 14. Prior to the hypotheses testing, we performed data cleanup and preparation tasks, as recommended by Tabachnick and Fidell, for a multivariate data analysis [44]. Following checks for univariate and multivariate outliers, we removed four cases that failed to meet cutoff values at *p* < 0.001 for Mahalanobis distance, Leverage, and Cook′s distance checks [45]. This left 356 cases for hypothesis testing.

Although we relied on previously developed scales to measures all four variables, items for two variables—PCT and the shorter AIP scale we used to measure procrastination tendencies—have not been used extensively or consistently among researchers. Therefore, we conducted a confirmatory factor analysis using SPSS AMOS v26.0 to assess reliability and convergent and discriminatory validity for all four variables. One item in the PCT scale—“I often have little control of what is happening at work” (reverse coded)—proved problematic for convergent validity and was dropped from the scale, leaving four items. The same was true for two items in the short version of the AIP, both of which were also dropped, leaving eight items. These two items were “*I am on time as most people I know*” (reverse coded) and “*Putting things off until the last minute has cost me money during the past year*.” The descriptive statistics, factor correlations, and reliability and discriminant validity results from the confirmatory factor analysis (CFA) appear in Table 1. All values met or exceeded the thresholds established in the literature for reliability, as well as convergent and discriminant validity [46]. The composite reliability (CR) values well exceeded the threshold of 0.70. Convergent validity was assessed by the average variance extracted (AVE), which equaled or exceeded 0.50 for each factor. Evidence of discriminant validity was demonstrated in that the square root of the AVE for each measure (shown in the diagonal in Table 1) exceeded the inter-construct correlations for that variable [47].

Next, because we used a single instrument to measure both the predictor and criterion variables in our model, we conducted a post-hoc Harman′s single factor analysis to test for common method variance (CMV). The purpose of this test is to assess the extent of CMV bias, so that the fitness of a model with each variable independently loading on its respective factor is a better fit than a model with a single factor that includes all variables [48]. A four-factor model revealed a better fit (CFI = 0.942, TLI = 0.935, RMSEA = 0.056) compared to the single factor model (CFI = 0.561; TLI = 0.525, RMSEA = 0.139). However, this was not the only method we used to control for CMV. We also followed good survey design practices, such as randomizing the order of appearance of the measures and varying scale ranges and response categories [49].

Lastly, we found no evidence of multicollinearity in the intercorrelations between predictors (see Table 1 or in variance inflation factor (VIF) values (VIF < 1.01) when the predictor variables were regressed upon each other [45]. The Durbin–Watson was 1.75, which was within range of 2.0 (between 1.5 and 2.5) for the independence of studentized residuals [50]. Prior to the hypothesis testing, the scale scores for each variable were summed, then averaged, and the scores that were used to calculate the interaction terms for the PCT and procrastination tendency variables were mean centered [51].

### 4.2. Tests of Hypotheses

Table 2 provides the results for the moderated mediation regression analysis of conditional indirect effects. In Hypothesis 1, we tested for direct effects between our predictor variable, workload demand, and our primary outcome variable, emotional exhaustion. The results for the overall model can be seen in the middle portion of Table 2. Workload demands had a significant positive effect on emotional exhaustion, which supported Hypothesis 1 (β = 0.213, *B* = 0.333, *t* = 4.89, *p* < 0.001). The top portion of Table 2 shows the results for the predicted direct path between workload demand and the mediating variable, PCT. Hypothesis 2 was also supported (β = −0.385, *B* = −0.327, *t* = −8.09, *p* < 0.001). Workload demands were negatively associated with PCT, suggesting that when workers experience higher workload demands, they feel less in control of their time.

**Figure 2:**
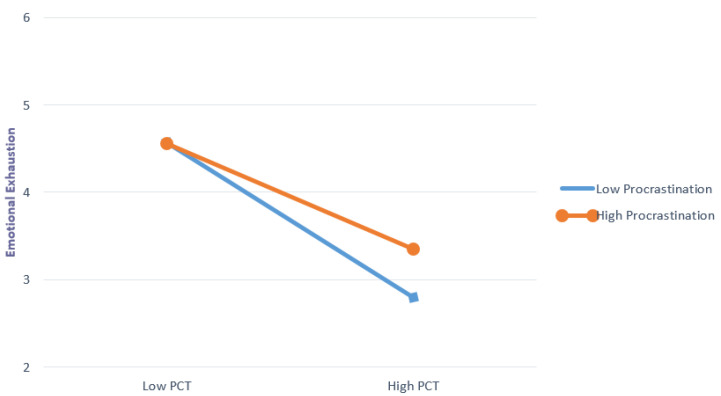
Interactive effects of procrastination and PCT on emotional exhaustion.

Turning now to the effect of PCT on emotional exhaustion, as seen in the full model results in the middle of Table 2, PCT was negatively associated with emotional exhaustion (β = −0.470, *B* = −0.864, *t* = −9.12, *p* < 0.001). However, the results from the moderation analysis, which examined the potential conditional effects (i.e., moderated mediation) of procrastination tendencies on PCT, were of primary concern for our hypotheses. The bottom portion of Table 2 shows the results for Hypothesis 3, in which we posited an interaction effect between PCT and procrastination tendencies. The interaction term was significant (β = −0.084, *B* = −0.202, *t* = 2.39, *p* < 0.02), demonstrating support for Hypothesis 3. The mitigating influence of PCT on emotional exhaustion was reduced by worker′s tendencies toward procrastination. This interaction effect is visualized in Figure 2. Figure 2 shows different values of emotional exhaustion for low and high values of PCT at different levels of procrastination. The figure illustrates that when a worker′s procrastination tendencies are high, high PCT fails to mitigate the levels of emotional exhaustion to the degree that it does when a worker′s procrastination tendencies are low.

Lastly, Hypothesis 4 is the critical hypothesis that tests the moderated mediated effects for the relationships proposed in the model. We posited that a conditional indirect effect of workload demands on emotional exhaustion arose from the PCT′s mediating influence, which was moderated by a worker′s procrastination tendencies. The results supporting Hypothesis 4 appear at the bottom part of Table 2. The bootstrap results, with 10,000 re-samples for simple slopes at low, medium, and high levels of procrastination, were significant for the moderated mediated path from PCT to emotional exhaustion, as indicated by the 95% confidence intervals that did not contain zero. Furthermore, the index of moderated mediation was equal to −0.066, with a bootstrap confidence interval from −0.116 to −0.020, which did not include zero. The index of moderated mediation tested the slope of the product of paths in the model as a function of the moderated mediation effect [43]. According to Hayes [43] (p. 425), when the bootstrap confidence intervals for the index do not contain zero, this single inferential test provides conclusive support for moderated mediation effects. In this case, because the index effect sign is negative, it indicates that the indirect effect is negatively related to the moderator, procrastination—that is, the mediation of the effect of workload demands on emotional exhaustion through a worker′s perceived control of time is lowered or undermined by a worker′s tendency to procrastinate, as we predicted in Hypothesis 4.

## 5. Discussion

Our aim was to investigate the potential mitigating role of the perceived control of time within a traditional job stressor and strain model. The JDC model has been utilized extensively for decades to understand and predict how elements in workplace environments may induce or prevent harmful effects of job demands on workers, including emotional exhaustion and stress. The role of control is central to the buffer hypothesis, but buffering effects related to workers′ control have proven both theoretically and empirically difficult to substantiate. We incorporated constructs from the time management literature, perceived control of time, and procrastination tendencies to propose and empirically test a moderated mediation model in which office workers′ control over time buffers the relationship between workload demands and emotional exhaustion, depending on workers′ tendency to procrastinate or failure to use time wisely. The findings supported the four hypotheses we proposed and tested.

The results from this study corroborate previous findings, yet extend the scope of the framework for examining the control variables and their impact on workers′ mental strain and stress outcomes. The current study replicates the positive association between workload demands and the psychological job strains found by others [8,10]. Theoretically, having a heavy workload is thought to induce anxiety and frustration among workers because it creates uncertainty, whether it be regarding their ability to perform the necessary tasks or what will happen to them if they cannot. However, not every worker who has a great deal to accomplish in a short amount of time experiences chronic anxiety or symptoms of burnout. In fact, having more to do than what one can accomplish in a given day is normal for most workers in today′s fast-paced environment [1]. We expand the framework of the JDC model by investigating how the perception of control over one′s time may influence job strain. The findings support the mediating influence of PCT on the relationship between work demands and work strain discovered by Claessens et al. [16]. Furthermore, we find support for a conditional effect of procrastination tendencies, an individual trait, on the PCT′s ability to mitigate emotional work demand outcomes. Individual traits have not been widely investigated in the JDC framework, and this study demonstrates the need to consider how these might explain why some individuals respond differently to equivalent work environment stressors than others.

This research also offers theoretical contributions to the job control and stress literature in organizational psychology. In the traditional JDC model, workload demands always lead to negative outcomes, which may be mitigated but never removed by giving workers more control. Our findings regarding the conditional effect of PCT on emotional exhaustion illustrate how the “fit” between job characteristics and perceptions of self-control may impact workers′ abilities to successfully adapt to even highly demanding work environments. To the extent that workers are effective at self-regulation, they may enjoy the arousal associated with meeting challenges and experience less stress in fast-paced work environments. This corresponds with the findings observed by researchers who have investigated adaptive or “active” forms of delay as opposed to procrastination as it has been traditionally viewed from the self-regulation failure framework [30,52]. Corkin, Yu, and Lindt [52] found that students who engaged in active delay were more likely to adopt mastery-approach behaviors, demonstrate the adaptive use of self-regulated learning strategies, such as relaxing or taking breaks, and report higher levels of self-efficacy than students who engaged in procrastination, who were more likely to adopt mastery-avoidance behaviors and report low self-efficacy and concerns about appearing inferior to others. Similarly, the “activation hypothesis” articulated in Karasek′s JDC model [23] predicts that high workload and high job autonomy will foster intrinsic motivation, learning, and personal growth, as individuals learn over time in spite of heavy work demands and develop a sense of competence at responding to overcoming challenges in their environment. Our conceptual model reflects Miller′s theory of the process through which control reduces stress more closely [35]. Her Minimax hypothesis posits that when individuals believe they are able to exert control over aversive events, they experience a reduction in uncertainty through their confidence in knowing that relief is available from a stable source—oneself. Confidence in self, or self-efficacy [53], has been linked to both PCT and procrastination. Macan, in his process model of time management, asserts that lower job-related tension and higher job satisfaction are not direct outcomes of effective time management practices per se, but instead are obtained indirectly through PCT and gaining “a sense of mastery over how one allocates one′s time” [15] (p. 382). Conversely, procrastinators, who are have low self-efficacy and are poor self-regulators [28,33], tend to perform more poorly and experience more stress as deadlines approach than non-procrastinators [32]. Over time, a pattern of successive failures may further undermine procrastinators′ self-confidence and ability to be intrinsically motivated and challenged by the task. In one of the few longitudinal studies of procrastination, stress, and performance, Tice and Baumeister [31] found evidence of a pattern of self-defeating behaviors, higher stress levels, poor performance, and low intrinsic motivation among students who procrastinated.

The current study reveals an association between PCT, procrastination, and stress, but more research is needed to understand the process and the psychological mechanisms through which they operate to produce a variety of outcomes. We acknowledge multiple limitations in our study. The study′s cross-sectional survey methodology limits our ability to demonstrate causal relationships. Experimental research could provide stronger inferences regarding direct and indirect effects of PCT on work stressors and strain. Longitudinal studies are also needed, especially with regard to understanding how procrastination tendencies and successive performance outcomes impact an individual’s confidence in their ability to control time resources and their self-efficacy beliefs. Organizational studies could also explore how PCT and procrastination and active delay operate conditionally and differentially among workers who exhibit different personal characteristics or perform work in different conditions. More studies are needed to examine the role of control variables in producing outcomes beyond those associated with job strain, such as job satisfaction and performance. In their study of R&D engineers, Claessens et al. [16] found that PCT was positively associated with both job satisfaction and performance among workers with high job autonomy. Future research could investigate the influence of PCT and procrastination in work conditions designed to promote personal control and job autonomy, such as flex schedules and telecommuting. Furthermore, our study involved a relatively small and generalized population of “office workers.” Future research is needed to determine if the relationships we found generalize across different job contexts or differ among subgroups of workers based on individual or job characteristics. Another interesting topic for future research is how actions of group members, such as failure to self-regulate, impacts stress levels and the performance of individual team members and/or groups. Lastly, our study did not consider the full range of time management variables, including goal setting, planning, and monitoring. Future studies are needed to understand how planning and other time-oriented strategies impact control and stress outcomes when workers face challenging demands and time pressures.

## 6. Conclusions 

In this study we proposed and tested a moderated mediation model, in which perceived control over time conditionally mitigates the positive relationship between workload demands and emotional exhaustion depending on a worker′s procrastination tendencies. The findings showed that high procrastination tendencies undermined the mitigating influence of the high perceived control of time on emotional exhaustion. These findings support the buffering effect of control in the traditional JDC model for work strain. Furthermore, this study demonstrates that individual traits, such as procrastination, may moderate the effects of perceived control over time on work outcomes.

## Figures and Tables

**Table 1:** Descriptive statistics and confirmatory factor validity and reliability measures for all variables.

Variables	Mean	SD	CR	AVE	1	2	3	4
1. Emotional Exhaustion	3.85	1.49	0.927	0.616	***0.785***			
2. Procrastination	2.17	0.76	0.888	0.500	0.402 ***	***0.707***		
3. Perceived Control Time	3.74	0.81	0.804	0.508	−0.702 ***	−0.658 ***	***0.713***	
4. Workload Demands	3.22	0.95	0.847	0.529	0.491 ***	0.097	−0.492 ***	***0.727***

Notes: *n* = 356; *** *p* < 0.001; emotional exhaustion was measured on a 7-pt scale with 7 high, rest on 5-pt scale with 5 high; SD = standard deviation; CR = composite reliability; AVE= average variance extracted; square root of average variance extracted listed on the diagonal in bold italics.

**Table 2:** Ordinary least squares regression results for direct, indirect, and conditional effects.

Outcome Variable: Perceived Control of Time						
Constant	1.479	0.161	9.20	0.001	1.163	1.795
Workload demands	−0.327	0.040	−8.09	0.001	−0.407	−0.248
Workload demands	−0.327	0.040	−8.09	0.001	−0.407	−0.248
PCT [Model *R* = 0.45; *R*^2^ = 0.20; MSE = 0.524, *F*(2353) = 45.05, *p* < 0.001]						
**Outcome Variable: Emotional Exhaustion**						
Constant	2.193	0.273	8.044	0.001	1.657	2.730
Workload demands	0.333	0.068	4.889	0.001	0.199	0.467
PCT	−0.864	0.095	−9.123	0.001	−1.050	−0.678
Procrastination	0.142	0.096	1.488	0.138	−0.046	0.330
PCT x Procrastination	0.202	0.084	2.397	0.017	0.036	0.368
EmEx [Model *R* = 0.68; *R*^2^ = 0.48; MSE = 1.208 *F*(5350) = 59.72, *p* < 0.001]						
Test of unconditional interaction PCT x Procrastination [∆*R*^2^ = 0.009, *F*(1, 350) = 5.75, *p* < 0.02]						
Conditional indirect effect of Workload demands -> PCT -> EmEx at different values of Procrastination						
Procrastination		*Boot indirect effect*		*Boot SE*	*Boot LLCI*	*Boot ULCI*
16th percentile −0.8006		0.3355		0.0592	0.2260	0.4574
50th percentile −0.1006		0.2858		0.0507	0.1924	0.3902
84th percentile 0.7994		0.2280		0.0481	0.1395	0.3285
Index of moderated mediation		−0.0662		0.0243	−0.1155	−0.0196

Notes: PCT = perceived control of time; EmEx = emotional exhaustion. Unstandardized regression coefficients are reported. Bootstrap sample size = 10,000. LLCI = lower limit confidence interval 95%; ULCI = upper limit confidence interval (bias-corrected bootstrap confidence intervals). PCT and procrastination are mean-centered.

## Appendix A

**Emotional exhaustion** ^a^
*Please indicate your level of agreement/disagreement with the following statements:*
I feel used up at the end of the workday.
I feel fatigued when I get up in the morning and have to face another day on the job.
Working all day with people is really a strain for me.
I feel frustrated by my job.
I feel like I′m at the end of my rope.
I feel burned out from my work.
I feel emotionally drained from my work.
I feel I′m working too hard on my job.
**Adult Inventory Procrastination** ^b^
*Indicate how you respond by selecting the rating alternative to each question that best fits your usual style.*
I am prompt and on time for most appointments. (R)
I often find myself running later than I would like to be.
I don′t get things done on time.
My friends and family think I wait until the last minute.
I get important things done with time to spare. (R)
I am not very good at meeting deadlines
I find myself running out of time.
When I need to be somewhere at a certain time my friends expect me to run a bit late. (R)
**Perceived Control of Time** ^b^
*Please indicate your level of agreement/disagreement with the following statements:*
I feel in control of my time.
I find it difficult to keep to my schedule because others take me away from my work. (R)
I feel that I have my work under control.
I feel confident in that I am able to complete my work on time.
**Quantitative Workload Inventory** ^C^
How often does your job require you to work very fast?
How often does your job require you to work very hard?
How often does your job leave you with little time to get things done?
How often is there a great deal to be done?
How often do you have to do more work than you can do well?
^a^ Anchored by 1 = strongly disagree and 7 = strongly agree
^b^ Anchored by 1 = strongly disagree and 5 = strongly agree
^C^ 1 = less than once per month or never; 2 = once or twice per month; 3 = once or twice per week; 4 = once or twice per day; 5 = several times per day
**(R) = reverse scored**
